# PERSONAL NARRATIVES OF AGING

**DOI:** 10.1093/geroni/igz038.2780

**Published:** 2019-11-08

**Authors:** Louise Taylor, Jan Bailey, Paul Kingston, Charlotte Eost-Telling

**Affiliations:** University of Chester, Chester, England, United Kingdom

## Abstract

This presentation reflects on self-written narratives from respondents to a mass observation directive, focusing on the experiences of growing older. Narrative methods are theoretically and methodologically diverse, and are helpful in social research to understand events or happenings in human lives. This data presents accounts from a heterogeneous sample in the form of self-penned responses. These experience-centred narratives bring stories of personal understanding into being by means of the first person description of past, present, future or imaginary experiences. This presentation will focus on the findings with reference to physical and mental impacts, both real and anticipated. We will also explore themes arising from the data including gender differences, age-cohort effects and stigma. The data can be used to inform Health and Social Care education and practice, particularly in co-producing appropriate person-centred services with older people.

